# Depression, C-reactive protein and length of post-operative hospital stay in coronary artery bypass graft surgery patients

**DOI:** 10.1016/j.bbi.2013.11.008

**Published:** 2014-03

**Authors:** Lydia Poole, Tara Kidd, Elizabeth Leigh, Amy Ronaldson, Marjan Jahangiri, Andrew Steptoe

**Affiliations:** a Department of Epidemiology and Public Health, University College London, 1-19 TorringtonPlace, London, WC1E 6BT; b Department of Cardiac Surgery, St. George’s Hospital, University of London, Blackshaw Road, London, SW17 0QT

**Keywords:** Depression, C-reactive protein, Coronary artery bypass grafting, Recovery

## Abstract

This study aimed to explore the role of C-reactive protein (CRP) in mediating the association between greater pre-operative depression symptoms and longer post-operative length of stay in patients undergoing coronary artery bypass graft (CABG) surgery. We used a sample of 145 elective CABG patients and measured depression symptoms using the Beck Depression Inventory (BDI) prior to surgery and collected baseline measures of CRP. Participants were followed up during their in-hospital stay to measure early (1–3 days post-surgery) and persistent (4–8 days post-surgery) CRP responses to surgery. We found that compared with participants with low depression symptoms, those with elevated depression symptoms (BDI > 10) prior to CABG were at increased odds of a hospital stay of greater than one week (OR 3.51, 95% CI 1.415–8.693, *p *= 0.007) and that greater persistent CRP responses mediated this association. Further work is needed to explore the exact physiological pathways through which depression and CRP interact to affect recovery in CABG patients.

## Highlight

• Elevated depression symptoms prior to CABG were associated with increased odds of extended hospital stays and post-operative CRP responses mediated this association.

## 1 Introduction

Depression is commonly experienced by patients undergoing coronary artery bypass graft (CABG) surgery (Blumenthal et al., 2003; Burg et al., 2003a; Oxlad and Wade, 2006; Pirraglia et al., 1999; Tully et al., 2008). Patients with pre-operative depression have been found to experience a host of poorer surgical recovery outcomes (Burg et al., 2003a) including longer post-operative hospital stays (Oxlad et al., 2006). Post-operative stay is a proxy measure of acute physical recovery as well as being an important indicator of recovery in the longer-term, as it has been found to be associated with hospital readmission (Hannan et al., 2003) and recurrent cardiac events (Connerney et al., 2001). Many clinical risk factors are established predictors of prolonged length of stay (Tu et al., 1995), though little attention has been paid to the role of inflammation.

Pre-operative C-reactive protein (CRP) levels have been shown to predict adverse outcomes in CABG patients (De Lorenzo et al., 2012), with results showing that those with elevated pre-operative CRP (⩾3 mg/l) had significantly greater risk of post-operative mortality. Elevated pre-operative CRP has also been shown to be associated with longer hospital stays after CABG (Perry et al., 2010). CABG surgery itself is associated with an acute inflammatory response and while the extent of the inflammatory response is thought largely to reflect the amount of trauma derived from the surgical procedure itself, it is associated with a host of clinical outcomes, both cardiac and non-cardiac in scope (Biglioli et al., 2003; Levy and Tanaka, 2003). An observational study of 29 cardiopulmonary bypass patients by Holmes and colleagues (Holmes et al., 2002) used a median split to compare outcomes between those patients who showed a heightened inflammatory response at four hours post-CABG, to those who did not. Findings showed that hyper-responders in interleukin (IL)-8, IL-6 and complement receptor-3 had greater risk of adverse clinical outcomes. Therefore, greater pre- and post-operative inflammatory markers have both been associated with poorer recovery following CABG surgery.

Depression is associated with an innate inflammatory response (Raison et al., 2006), and a meta-analysis by Howren and colleagues has shown depressive symptoms to be positively associated with CRP, IL-1 and IL-6 in both clinical and community samples (Howren et al., 2009). A more recent meta-analysis by Dowlati and colleagues (Dowlati et al., 2010) has supported the role of pro-inflammatory markers in depression, in particular IL-6 and tumor necrosis factor (TNF)-α. Epidemiological evidence for the directionality of the depression–inflammation relationship is mixed, with some studies suggesting that depression precedes inflammation (Stewart et al., 2009), while others show that inflammation precedes depression (Gimeno et al., 2008). Many investigators argue that there is a bidirectional association between depression and inflammation (Maier and Watkins, 1998; Matthews et al., 2010).

Only one study to date has assessed the association between depression and inflammation in CABG patients (Yang et al., 2012). These authors studied 232 patients undergoing CABG surgery and found that higher pre-operative high sensitivity (hs)-CRP was predictive of depression symptoms up to six months following surgery, after controlling for covariates. However, little is known about the direction of the causal relationship between depression and CRP for predicting CABG recovery. The aim of this study was to prospectively assess the relationship between pre-operative depression symptoms and length of post-operative stay, and to examine the extent to which any association could be moderated by inflammation both pre- and post-surgery. Specifically, we hypothesised that greater depression symptoms would be associated with longer hospital stays, and that the effect of depression symptoms on length of stay would be mediated by in-hospital hs-CRP.

## 2 Methods

### 2.1 Participants

**Fig. 1:**
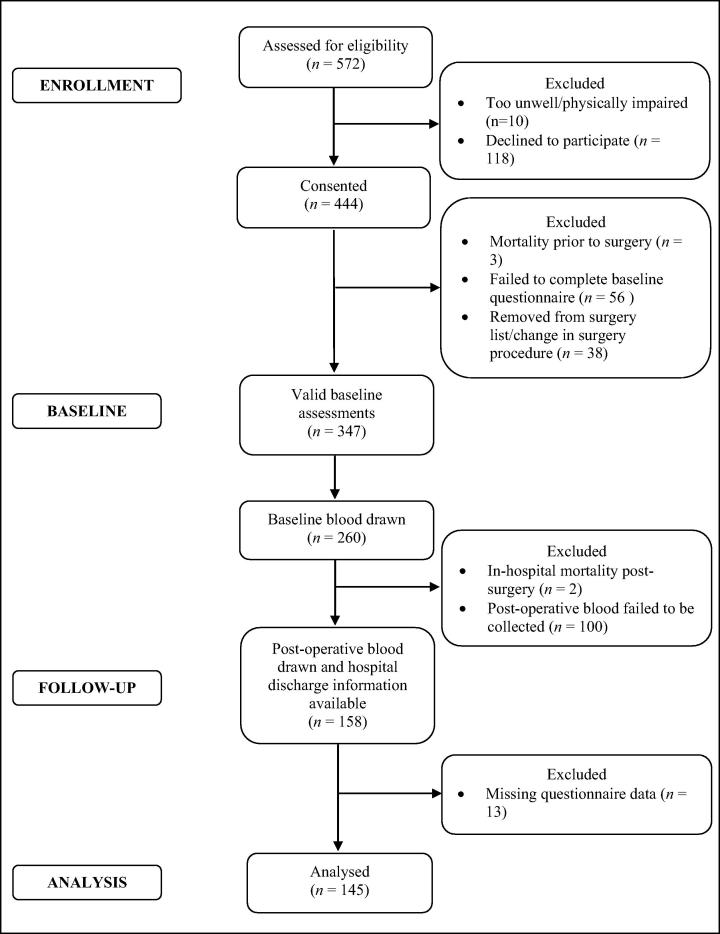
Flow diagram of participant recruitment and attrition.

The study uses data collected in the Adjustment and Recovery after Cardiac Surgery (ARCS) Study. The recruitment and retention of participants into the ARCS Study is displayed in Fig. 1. Briefly, 347 patients were recruited to the study, and pre-surgery blood samples were obtained from a subsample of 260 participants (see Fig. 1). Participants included in these analyses were the 145 CABG surgery patients (mean age: 67.49 ± 9.09 years, 10.3% females) with complete data for all variables at baseline and follow-up, including covariates. Compared with the participants who were included in these analyses (*N* = 145), the participants who had missing follow-up blood data and missing questionnaire data (*N* = 115) were more likely to have had a greater number of grafts (*t *= −2.65, *p* = 0.009) and a lower EuroSCORE (*t *= 2.02, *p *= 0.045), but otherwise did not differ on any other clinical or demographic variable. Participants were consecutively recruited from a pre-surgery assessment clinic at St. George’s Hospital, London. The baseline assessment took place on average 31 days before patients’ surgery when they came to the hospital for their pre-assessment clinic appointment. Inclusion criteria permitted only patients who were undergoing elective CABG surgery or CABG plus valve replacement to participate. CABG surgery was defined to include both on-pump and off-pump surgical procedures. In addition, participants had to be able to complete the questionnaires in English, and be 18 years or older. Participants who were too unwell to participate or who were physically impaired (e.g. visual impairment) were excluded, based on the advice of the pre-assessment clinic nurses. However, we did not specifically exclude participants because of concurrent mental or physical illness. All procedures were carried out with the written consent of the participants. Ethical approval was obtained from the South West London research ethics committee.

### 2.2 Measures

#### 2.2.1 Predictor: depression symptoms

The Beck Depression Inventory (BDI) (Beck et al., 1988) was used to measure depression symptoms at baseline. It is a 21-item questionnaire which asks the respondent to reflect on how they have been feeling over the past 2 weeks. Ratings were summed, with higher scores indicating greater emotional disturbance, with a range of 0–63 (Cronbach’s *α* = 0.85). A binary variable was generated according to accepted cut-offs: a score of 0–10 indicating no depression and 11–63 suggesting mild to severe depression.

#### 2.2.2 Mediator: hs-CRP immunoassays

To assess inflammatory activity, a 20 mL blood sample was drawn into a serum separator tube by venepuncture from the forearm at baseline. During the post-operative in-hospital stay, blood samples were drawn on approximately day 1 and 4 after surgery. Due to the variability in collection day for the post-operative bloods, two summary scores were created. The first summary score used the mean for the hs-CRP values on days 1, 2 and 3 post-surgery and the second used the mean for the hs-CRP values on days 4, 5, 6, 7 and 8 post-surgery. These scores were termed ‘early’ and ‘persistent’ responses. Blood was allowed to clot and centrifuged for 10 min at 3000 rpm. The resulting serum was frozen at −80 °C until batch analysis at a later date. Assays were performed by Dr. David Gaze at St. George’s Healthcare NHS Trust, using commercial automated immunoassay on the Immulite 1 (Siemens Healthcare Diagnostics, Frimley, Surrey). The minimal detectable dose was 0.01 mg/dL, the upper limit of detection was 15 mg/dL and the intra-assay CV range was 4.2–6.4%.

#### 2.2.3 Outcome: length of stay

Length of post-operative hospital stay was collected from clinical records. Length of stay is a proxy marker of clinical recovery, with those participants experiencing the poorest recovery and the greatest in-hospital complications, expected to have the longest hospital stays after CABG. The policy at the hospital during this period was to discharge patients within 7 days of CABG providing there were no complications, and this policy remained in force throughout the period of data collection.

#### 2.2.4 Covariates: cognitive, clinical and sociodemographic measures

The Montreal Cognitive Assessment (MoCA) (Nasreddine et al., 2005) was administered at baseline. It was developed as a brief measure of mild cognitive impairment covering eight cognitive domains: visuospatial awareness, executive functioning, short-term memory, attention, concentration, working memory, language and orientation to time and place. A maximum of 31 points was awarded, including an extra point being awarded to participants with ⩽12 years of education.

Cardiovascular history, clinical factors during admission and management were obtained from clinical notes. Clinical risk was assessed using the European System for Cardiac Operative Risk Evaluation (EuroSCORE) (Roques et al., 2003). EuroSCORE is a composite measure of procedural mortality risk based on 17 factors comprising patient-related factors (e.g. age, sex), cardiac-related factors (e.g. unstable angina, recent MI) and surgery-related factors (e.g. surgery on thoracic aorta). Items were scored in accordance with the ‘logistic EuroSCORE’ method to generate a percentage mortality risk estimate; further details of the scoring method can be found on the EuroSCORE website (www.euroscore.org/logisticEuroSCORE.htm). In addition, the number of grafts a participant received and whether they underwent cardiopulmonary bypass (yes/no) were also recorded. History of diabetes was taken from medical notes, with participants being categorised according to their treatment status: none, diet, oral hypoglycaemic drugs or insulin. Participants were asked to report any longstanding illnesses prior to surgery; responses were counted to compute a chronic illness burden variable to capture the number of illnesses a participant had in addition to their coronary artery disease. Prescribed medication use was recorded, including use of antidepressants and statins. Smoking was measured as a binary variable (current smoker/non-smoker). Body mass index (BMI) was assessed at the pre-operative clinic appointment and calculated using the standard formula (kg/m^2^).

### 2.3 Statistical analysis

Associations between variables were assessed using Pearson’s correlations for continuous data and *t*-tests and chi-square tests for categorical variables. To test the association between baseline depression and length of hospital stay, we analysed baseline depression as a binary variable using the BDI standard cut-off of 10, and modelled associations between pre-operative depression and post-operative length of stay using logistic regressions. Since data on length of post-operative hospital stay were skewed, a binary cut-off of 7 days was implemented. This approach was deemed appropriate given it is a clinically pertinent indicator of recovery, with St. George’s Hospital guidelines aiming for all patients to be discharged within 7 days of CABG surgery. Linear regressions were performed using the continuous BDI scores to corroborate the findings found with the binary BDI variable; due to the length of stay data being skewed, this variable was log-*n* transformed prior to use in these models. We have chosen to use the results using the binary outcomes as the primary analyses here since they are more clinically meaningful. We included covariates that might potentially relate to the outcomes including: age, sex, BMI, smoking status, diabetes status, cardiopulmonary bypass, number of grafts, chronic illness burden, use of antidepressant medication, EuroSCORE and cognitive function (MoCA). Since age and sex and CABG in isolation are included in EuroSCORE, these covariates are not included in the fully adjusted models so as to avoid double adjustment. Results are presented as adjusted odds ratios with 95% confidence intervals (CI). Models adjusting for use of statin medications were also performed, but are not reported here since the results did not change; statin records were missing for 9 participants.

We tested the association between hs-CRP and length of stay using logistic regression models. Post-operative hs-CRP responses to surgery were modelled as change scores, subtracting the baseline hs-CRP value from the post-operative value (for both the early and persistent samples); larger change scores indicate a greater response from baseline to follow-up. There were 44 participants with missing early hs-CRP samples so the *N* in these analyses is 101. Baseline hs-CRP was also included in the models as a separate covariate to assess the relative contribution of both pre- and post-operative hs-CRP values on length of stay. Separate models were also performed on baseline hs-CRP to predict length of stay. Additional adjustments were made for age, sex, BMI, smoking status, diabetes status, cardiopulmonary bypass, number of grafts, EuroSCORE, chronic illness burden, use of antidepressant medication, and MoCA, and are presented as adjusted odds ratios with 95% CI. Again, since age and sex and CABG in isolation are included in EuroSCORE, they were not included in fully adjusted models.

The association between depression and hs-CRP was assessed using both *t*-tests and linear regression models. Finally, in order to directly test mediation, the Sobel test was implemented, using the method set out by Preacher and Hayes (2008, 2004). This method examines the mediation of the predictor and outcome variables, and tests the significance of the indirect effect in the model. The bootstrapping technique advocated in this method was also employed. The model included baseline binary BDI score as the independent variable and binary length of stay as the dependent variable, with hs-CRP persistent change entered as the mediator. Covariates included BMI, smoking status, diabetes status, cardiopulmonary bypass, number of grafts, EuroSCORE, chronic illness burden, antidepressant use, MoCA, and baseline hs-CRP. All analyses were conducted using SPSS version 21.

## 3 Results

Table 1 summarises the characteristics of the participants at baseline, prior to CABG surgery. The sample had an age range between 45 and 87 years, was predominantly male (89.7%) and overweight (BMI > 25 = 83.4%). The majority of participants were hypertensive, and approximately a quarter of patients were diabetic. The majority of participants had on-pump cardiopulmonary bypass surgery in isolation. The mean length of post-operative hospital stay was 8 days, with a range of 4–66 days. The majority of participants were within the normal range for depression symptoms on the BDI, however 45 (31.0%) participants scored >10 at baseline. Depression symptoms (BDI > 10) were associated with younger age (*t*(143) = 3.96, *p < *0.001) and smoking (*x*^2^ = 6.21, *p* = 0.013). No associations were found between BDI scores and use of statin or antidepressant medication. Longer hospital stays were associated with smoking (*x*^2^ = 6.21, *p* = 0.013), use of antidepressant medication (*x*^2^ = 7.62, *p* = 0.006), and CABG in isolation (*x*^2^ = 15.41, *p* < 0.001). As shown in Table 2 46.7% of patients with elevated BDI scores had hospital stays of 7 days or longer, compared with 24.0% of patients with no depression symptoms (*x*^2^ = 7.45, *p* = 0.006). Using the 45 participants with elevated depression symptoms (BDI > 10), we compared those with stays of 7 days or less with those with stays of 7 days or more. Results showed that the depressed extended stay participants were more likely to be on antidepressants than the depressed non-extended stay participants (*x*^2^ = 5.02, *p* = 0.025). While only 4 participants were using antidepressants in our depressed group, all of them were in the extended stay group. However, the mean BDI values did not significantly differ between the two groups (*t*(43) = −1.60, *p* = 0.117).

### 3.1 The association between depression symptoms and hs-CRP and length of stay

Table 3 shows the results from two separate logistic regression models predicting length of post-CABG stay. Model 1 shows that compared with the no depression symptoms group, patients in the mild to severe depression group had a 3.5 times greater odds of a hospital stay of longer than one week (*p = *0.007) in fully adjusted models. EuroSCORE (odds ratio = 1.24, CI = 1.062–1.437, *p* = 0.006) and use of antidepressant medication (odds ratio = 5.60, CI = 1.071–29.290, *p = *0.041) were the only other significant predictors in the fully adjusted model. Additional adjustment for use of pre-operative statins did not alter the results (results not presented here). These results were confirmed using the continuous BDI scores at baseline (*t* = 2.19, *p* = 0.030) entered into fully adjusted linear regression models to predict log transformed length of stay. Model 2 in Table 3 presents the logistic regression results for change in early hs-CRP predicting length of stay. The results show that for every unit increase in early hs-CRP change there was a 1.0% increase in the odds of an extended hospital stay in fully adjusted models (*p* = 0.030). Use of antidepressants (odds ratio = 6.26, CI = 1.007–38.884, *p* = 0.049) and lower MoCA scores (odds ratio = 0.86, CI = 0.740–0.995, *p* = 0.043) were the only other significant predictors of length of stay in this model. Additional adjustment for use of pre-operative statins did not alter the results (results not presented here).

Model 3 in Table 3 presents the logistic regression results for change in persistent hs-CRP predicting length of stay. The results show that for every unit increase in persistent hs-CRP change there was a 1.3% increase in the odds of an extended hospital stay in fully adjusted models (*p *= 0.002). There were no other significant predictors in this model. Again, additional adjustment for use of pre-operative statins did not alter the results (results not presented here).

Separate logistic regression models were also performed to assess baseline levels of hs-CRP on length of stay; however, baseline hs-CRP was found to be a non-significant predictor in fully adjusted models (odds ratio = 1.00, CI = 0.959–1.042, *p = *0.980).

### 3.2 The association between depression symptoms and pre- and post-operative CRP

**Fig. 2:**
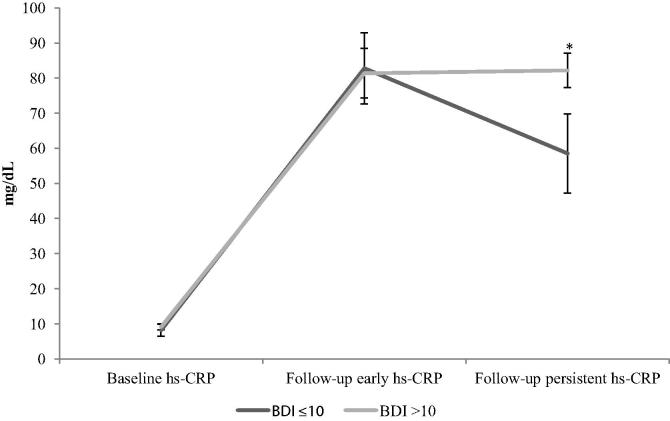
Change in hs-CRP from pre-operative to post-operative early and post-operative persistent periods according to BDI status. Bars are standard errors of the mean. *^∗^t* = −2.50, *p* = 0.026.

*T*-Tests were used to demonstrate the difference in hs-CRP levels at baseline and follow-up between depressed and non-depressed participants. These results are displayed in Table 4 and reveal a significant difference according to depression status on late hs-CRP responses. Linear regression models were performed to examine the cross-sectional relationship between BDI scores (*t* = 0.19, *p* = 0.851) and hs-CRP levels measured pre-operatively, with no significant association found. Nor was baseline BDI score (*t = *0.03, *p = *0.977) associated with change in early hs-CRP responses. However, in models to predict change in persistent hs-CRP responses, baseline BDI score (*t = *2.62, *p = *0.010) was a significant predictor in fully adjusted models (see Table 5). The only other significant predictors in this model were EuroSCORE (*t = *2.83, *p = *0.005) and a greater number of grafts (*t = *2.33, *p = *0.021). The change in hs-CRP over time according to status on the BDI is illustrated in Fig. 2 showing a similar acute inflammatory response to surgery in both BDI groups; however, the depressed participants (hsCRP mean = 82.19, standard deviation 75.51 mg/dL) showed a significant continued elevation in hs-CRP in the persistent sample compared to the non-depressed participants (hsCRP mean = 58.51, standard deviation 49.30 mg/dL) (*t* = −2.50, *p* = 0.026).

### 3.3 Mediation model of baseline depression, persistent hs-CRP change and length of stay

**Fig. 3:**
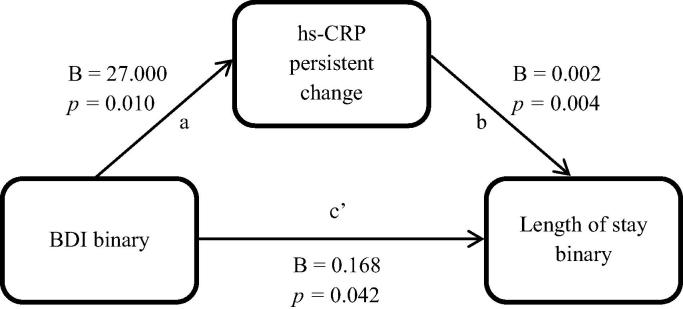
Mediation model of baseline BDI score and length of stay through change in persistent hs-CRP.

Since baseline BDI scores were not associated with early hs-CRP change, mediation models were only performed using persistent hs-CRP change. A Sobel test was used on the data, where pathway *c*’ represents the direct effect after controlling for the mediator, and pathway *ab* represents the total indirect pathway of the mediator (see Fig. 3). The indirect pathway was significant in this model (*B* = 0.05, SE = 0.028, 95% CI = 0.012–0.128), confirming change in persistent hs-CRP to be a significant mediator in this model. Partial mediation was shown such that the direct effect remained significant after persistent hs-CRP change was taken into account.

## 4 Discussion

In this study we aimed to examine the role of CRP in the relationship between pre-operative depression and length of post-operative hospital stay in patients undergoing elective CABG surgery. We were able to show that patients with elevated depression symptoms on the BDI were at increased odds of an extended hospital stay, and that this association was partially mediated by a greater change in CRP from baseline values to 4–8 days post-surgery. This study is the first to our knowledge which has prospectively examined the association between depression and CRP and their impact on physical recovery in a CABG population.

Our findings are consistent with previous research which has reported on the prevalence of depression symptoms prior to CABG surgery. In our sample, one third of participants had elevated scores on the BDI pre-operatively. Estimates of depression rates before CABG surgery have varied from between 14.3% and 43.1% (Blumenthal et al., 2003; Burg et al., 2003a; Oxlad and Wade, 2006; Pirraglia et al., 1999; Tully et al., 2008). The variability in these estimates are likely attributable to differences in the measurement of depression symptoms (e.g. questionnaire or clinical interview), different cut-off scores being implemented on questionnaire measures, differences in the timing of depression measurement in relation to surgery and clinical and demographic differences between samples.

Other studies have examined the detrimental effect of pre-operative depression for post-CABG surgical recovery. Burg and colleagues (Burg et al., 2003a) found that patients who were depressed pre-surgery had higher levels of medical complications during the six months following surgery, and were more likely to report poor quality of life and worse recovery. Moreover, 2 years after surgery, pre-surgery depression was linked to greater risk of death (Burg et al., 2003b). The role of depression on length of hospital stay has been previously examined by Oxlad and colleagues (Oxlad et al., 2006) who reported similar findings to the associations presented here, in that greater pre-operative depression symptoms were associated with longer hospitalisation. Among the depressed participants, we found only one difference in the clinical and demographic profile between those having extended stays compared to those discharged within a week of surgery, namely the extended stay participants were more likely to be taking antidepressants. However, there was no difference in mean BDI scores between these groups, which suggests that depression severity cannot account for the difference in length of stay. We addressed the possible influence of antidepressant use by using this as a covariate in fully adjusted models. Mechanisms underlying the association between depression and poor surgical recovery have received little attention previously.

Pre-operative CRP has been associated with poorer outcomes in CABG surgery patients, however the role of post-operative surgical responses has been less well studied. In our analyses we were able to show that change in CRP responses from pre-CABG to measures taken in the early and persistent post-operative phases were associated with increased odds of an extended length of stay. However, pre-operative measures of hs-CRP were not associated with length of stay. Our results are in line with a study of 29 participants by Holmes and colleagues (Holmes et al., 2002) who found that those with the largest inflammatory response 4 h post-CABG were at greatest risk of adverse clinical outcomes. More work is needed to tease apart these temporal effects of CRP in greater detail using larger samples and a variety of physical recovery endpoints. In addition, since length of stay is a marker of short-term recovery, future work is needed to address whether the same effects will be borne out in the longer term.

Our results show that while baseline levels of CRP were unrelated to depressive symptoms, at the post-surgery assessment a discrepancy occurred with depressed participants failing to return to basal levels at the same rate as non-depressed participants. This result suggests that depression symptoms moderate the body’s ability to self-regulate following cardiac surgery. An analogous finding was reported by Shaffer and colleagues in a sample of 163 acute coronary syndrome patients (Shaffer et al., 2011). These authors reported that elevated depression symptoms predicted a lesser decrease in CRP across the month following the cardiac event, which the authors describe as reflecting a poorer remission in the underlying inflammatory process. Our findings are the first to our knowledge that have explored this effect in CABG surgery patients. Our results suggest that pre-operative depression in some individuals promotes persistent inflammation beyond the period that might be expected by the physical trauma caused by the surgery itself. This persistent inflammation in turn might lead to phenomena that produce more extended hospital stays, such as infection or slower wound healing. This hypothesis requires further testing using other post-operative recovery endpoints to corroborate our findings using length of stay.

We have also provided evidence that change in hs-CRP from baseline to the 4–8 days after surgery partially mediated the relationship between pre-operative depression and length of post-CABG hospital stay. Other mechanisms are also likely to play a role alongside the inflammatory pathway. For example, it is possible that behavioural pathways such as physical activity, diet and medication adherence contribute to the relationship between pre-operative depression and recovery following CABG surgery, since previous work has found such factors to be relevant to both depression and cardiac health (Rieckmann et al., 2011; Whooley et al., 2008). While smoking and BMI were included in our fully adjusted models, further attention should be paid to the role of other lifestyle factors in future work on CABG patients.

Length of stay is likely to be affected by a variety of clinical and non-clinical factors and therefore we would emphasise that this outcome measure is only a proxy marker of physical recovery. Nevertheless, the fact that depression negatively impacted length of stay remains a clinically significant result. For example, the cost of hospital stays for CABG surgery have been estimated to be approximately £1500 for every day spent in intensive care (The Lancet, 2010) and £264 for every day spent on the general ward (Department of Health, 2012). In the ARCS study the surgery was carried out in a public hospital, where issues such as insurance cover and cost do not influence length of stay.

There are several strengths to our study. The longitudinal design of the ARCS study allows for the temporal relationship between depression, CRP and length of stay to be analysed. Moreover, the repeated assessment of CRP has allowed us to test the contribution of pre-operative and post-operative inflammation. In addition, the ARCS study examined patients undergoing CABG at a single hospital and therefore removes the influence of inter-hospital variation in discharge policy. However, there are also a number of limitations. Firstly, we have relied on a questionnaire measure of depression symptoms, which restricts us from generalising our results to clinically depressed samples. Secondly, while we have used length of stay as a proxy measure of physical recovery in the sample, we must also acknowledge that other, non-medical, factors are also likely to play a role in determining length of stay such as social housing constraints; such confounders were not able to be taken into account in our analyses. Lastly, there was a preponderance of male participants in the ARCS study with 10.3% of the sample being female. This male majority is characteristic of the CABG surgical population more generally, with men more likely to receive a revascularisation procedure than women in the UK. For example, in the ARCS study 444 patients were invited to participate, out of these only 66 patients (14.9%) were female. Therefore, the total number of women included in these analyses only slightly underrepresents the targeted clinical population. However, we included sex in all analyses as a covariate in order to address this issue. The findings we have reported have clinical implications regarding the treatment and screening of depression in a CABG population. However, more work is needed in order to investigate the best ways to approach the treatment of depression in this patient group.

In conclusion, we have found that pre-operative depression symptoms were associated with longer post-operative hospital stays in patients undergoing CABG surgery, and that this association was mediated by changes in CRP from pre-operative to post-surgery levels. Further work is needed in order to understand the processes through which depression and CRP interact to affect cardiac recovery.

## Funding

This research was funded by the British Heart Foundation.

## Conflict of interest

None.

## Figures and Tables

**Table 1:** Demographic, clinical and depression characteristics of the sample at baseline (*N* = 145).

Characteristic	Mean ± SD or *N* (%)
Age (years)	67.49 ± 9.09
Female	15 (10.3)
BMI (kg/m^2^)	28.99 ± 4.24
Smoker	13 (9.0)
Statin medication	120 (88.2)⁎
Antidepressant medication	10 (6.9)
Cognitive function (MoCA)	25.39 ± 3.64

*Co-morbidities*	
Chronic illness burden	0.48 ± 0.71
Diabetes	35 (24.1)
Hypertension	119 (82.1)
Pulmonary disease	10 (6.9)
Neurological disorder	11 (7.6)
Extracardiac arteriopathy	13 (9.0)

*Clinical factors*	
Logistic EuroSCORE (%)	4.21 ± 2.79
CABG in isolation	113 (77.9)
Number of grafts	3.12 ± 1.14
On-pump	112 (77.2)
Baseline hs-CRP (mg/dL)	8.46 ± 9.46
Follow-up ‘early’ hs-CRP (mg/dL)	82.39 ± 58.07⁎⁎
Follow-up ‘persistent’ hs-CRP (mg/dL)	65.86 ± 59.45
Length of post-operative hospital stay	
⩽7 days	100 (69.0)
>7 days	45 (31.0)

*Depression symptoms*	
Total BDI score	9.13 ± 7.04
Depression binary	
None (0–10)	100 (69.0)
Mild to Severe (11–63)	45 (31.0)

⁎ *N* = 136.

⁎⁎ *N* = 101.

**Table 2:** Cross-tabulation of depressed and non-depressed participants by length of stay (LoS).

	LoS < 7 days	LoS > 7 days	Total
*BDI ⩽ 10*			
*n*	76	24	100
% within ⩽ 10	76.0	24.0	100
*BDI > 10*			
*n*	24	21	45
% within > 10	53.3	46.7	100.0
Total	100	45	145

**Table 3:** Depression symptoms and hs-CRP change predicting length of post-operative hospital stay.

Model		OR	95% CI	*p*
*BDI score*				
Age and sex adjusted	None	1 (Reference)	–	–
	Mild to severe	3.813	1.660–8.760	0.002
Fully adjusted⁎	None	1 (Reference)	–	–
	Mild to severe	3.508	1.415–8.693	0.007

*hs-CRP early change (baseline to 1–3 day follow-up)*				
Age and sex adjusted	pg/mL	1.008	1.000–1.016	0.042
Fully adjusted⁎⁎	pg/mL	1.010	1.001–1.020	0.030

*hs-CRP persistent change (baseline to 4–8 day follow-up)*				
Age and sex adjusted	pg/mL	1.013	1.006–1.020	<0.001
Fully adjusted⁎⁎	pg/mL	1.013	1.005–1.021	0.002

⁎ Fully adjusted model: smoking status, BMI, diabetes, cardiopulmonary bypass, number of grafts, EuroSCORE, chronic illness burden, antidepressant use and MoCA.

⁎⁎ As fully adjusted model 1, with additional adjustment for baseline hs-CRP.

**Table 4:** Differences in hs-CRP responses according to depression status.

hs-CRP (mg/dL)	BDI ⩽ 10/>10	*N*	Mean	SD	*t*	*p*
Baseline	Not depressed	100	8.17	8.40	−0.548	0.585
	Depressed	45	9.10	11.56		
1–3 days post-surgery	Not depressed	71	82.81	59.50	0.111	0.912
	Depressed	30	81.39	55.52		
4–8 days post-surgery	Not depressed	100	58.51	49.30	−2.250	0.026
	Depressed	45	82.19	75.51		

**Table 5:** Depression symptoms predicting change in hs-CRP from baseline to the persistent post-operative period.

Model	*B*	SE	*β*	*p*
Smoking status	21.82	17.2	0.11	0.207
BMI	1.41	1.16	0.10	0.228
Number of grafts	9.97	4.28	0.20	0.021
Cardiopulmonary bypass	8.61	11.42	0.06	0.453
Diabetes	0.92	7.02	0.01	0.896
EuroSCORE	5.00	1.76	0.24	0.005
Chronic illness burden	−1.94	9.16	−0.02	0.832
Antidepressant use	19.37	18.89	0.09	0.307
MoCA	1.85	1.27	0.12	0.149
Baseline hs-CRP	0.80	0.50	0.13	0.110
Baseline binary BDI	27.00	10.29	0.22	0.010
